# “It’s more than milk, it’s mental health”: a case of online human milk sharing

**DOI:** 10.1186/s13006-021-00445-6

**Published:** 2022-01-04

**Authors:** Amanda J. Wagg, Alexander Hassett, Margie M. Callanan

**Affiliations:** 1 Anglia Ruskin University, Cambridge, England; 2 Salomons Centre for Applied Psychology, Canterbury Christ Church University, Kent, UK

**Keywords:** Human milk sharing, Online milk sharing, Online social support groups, Online support, Tangible online support

## Abstract

**Background:**

Milk sharing is not a new concept and occurs today via regulated human milk banks and unregulated online milk sharing groups. Exploring and understanding how, and why, mothers use these peers to peer milk sharing groups, is a vehicle to understanding how breastfeeding mothers can be tangibly supported online, adding to the literature on peer milk sharing, from a recipient’s perspective. This research presents a single case example of an online breastfeeding support group use, through one mother’s experiencing of seeking human donor milk.

**Method:**

This is a qualitative, exploratory study observing the attitudes, thoughts, and feelings of one mother who is seeking human donor milk through online groups. A single key case was identified, and the participant was asked to document thoughts and feelings as she searched for milk online. A telephone interview was conducted after two months, and the online page activity from the Human Milk for Human Babies Facebook group was captured for the week following the interview. The results were presented in a chronological and linear analytical approach adopting pattern matching.

**Results:**

‘Abbi’ is a mother who has Polycystic Ovary Syndrome and subsequent low milk supply and sought donor breastmilk online. Online support groups introduced her to donor milk sharing, which not only supported her breastfeeding but supported her own mental health. Abbi talks of the need to build a trusting relationship with her donor, due to the lack of regulation, and the positive impact it had for her and ‘Lucas’, her baby.

**Conclusion:**

Considering milk sharing groups simply as tangible online support ignores the complexities around Abbi’s decision to use human donor milk. Peer milk sharing online is an option for mothers, but it is surrounded by stigma amongst other mothers, professionals, and even within pro breastfeeding support groups.

## Background

The United Kingdom (UK) has some of the lowest breastfeeding rates in the world [1], and because of the known benefits for mothers, babies, and society, breastfeeding is a public health priority [2, 3]. There is a need to scale up, and monitor, breastfeeding interventions aimed at supporting breastfeeding mothers [4], and human milk banks, and peer-to-peer sharing of human milk online, are both interventions that support women to provide breastmilk for their babies. This tangible support avenue may be considered taboo but is considered by many mothers.

The aim of this research was to produce a single case example of a tangible online breastfeeding support group, demonstrated through one mother’s experiences of seeking peer to peer breastmilk online. This research explores how and why the online support group was used; and how this tangible support might potentially buffer breastfeeding stressors. This research aims to add to the body of literature on online support groups, and further explore how to best support breastfeeding mothers through online platforms.

### The history of milk sharing

In eighteenth century Britain ‘Wet nursing’ as a paid profession, was a popular appointment by wealthy families and in Victorian Britain, many wet nurses continued to work in the homes of wealthy women [5]. However, wet nursing became much less popular in late nineteenth century for a number of possible reasons; the introduction of modified cow’ milk formula, journalists publishing articles about the high mortality rates of infants who were wet nursed, the alleged spreading of disease by wet nurses, and reports of wet nurses abandoning their own children [5]. It could be argued that this was the start of the stigma surrounding milk sharing in the UK today.

Wet nursing is not unique to the UK and has existed in cultures around the world for centuries. Mentioned in the Old Testament, writings from ancient Egypt, Greek mythology, and ancient Roman texts, show that wet nursing has been practiced by many cultures to ensure the survival of the child [6]. In eighteenth century France approximately 90% of infants being wet nursed [7]. In Europe, wet nursing reportedly continued throughout World War one [8], and in the United States, it was commonplace for enslaved black women to wet nurse the white children with whom they lived [7]. The historical relationship between wet nursing and slavery may also contribute to the stigma surrounding wet nursing practice.

In many cultures, especially in the UK, wet nurses often took on more roles in childcare other than simply nursing, developing a relationship with the child and family. This is also seen in contemporary Vietnamese family structure, where ‘wet nurse’ translates to mother, and in Islam where ‘milk kinship’ is practiced in many Arab countries, whereby the child would have a second family to support them and breastfeed them if harm came to the biological parents [9].

In developed countries there is a social discomfort around milk sharing [10], with suspicious around the transmission of infection [11, 12], concerns around the donors’ lifestyle habits [13], and lack of regulation [14]. Human milk, in its unpasteurised raw state, could pose health risks similar to any other food stuffs [15, 16].

### UK Milk banks

UK milk banks emerged around 1985 as non-profit organisations to offer pasteurised human milk. In the UK today there are 18 regulated human milk banks, mostly sited in NHS hospitals, all affiliated with the Associations of Milk Banks, and all adhere to the National Institute for Health and Care Excellence (NICE) Guidance for Milk Banking: service information [17]. However, it is estimated that over 130,000 mothers participate in online milk-sharing networks [18], with tens of thousands of milk sharing exchanges worldwide are facilitated through websites annually [19] and considered by 25% of women [15]. In fact, if breastfeeding was not possible then donor human milk should be offered before a modified cow’s milk [2].

### Human milk sharing groups

Human milk for human babies’ (HM4HB) is an online breastmilk sharing site, on Facebook [20], and runs in almost 30 countries. It links women that have breastmilk to donate with women that would like to receive it, with no monetary gain. These milk sharing sites appear to offer tangible support facilitated through an online platform. They appear to offer little emotional and esteem support, just tangible support through informational posts purely related to milk sharing.

HM4HB operates outside of the regulated milk banks offering tangible support to breastfeeding mothers, with the donor appearing to have purely altruistic reasons to help women with an insufficient milk supply. The donor has often expressed milk for later use but for several reasons does not go onto to use it, nor want to see it wasted [19]. There are medical reasons why a mother might struggle with sufficient milk supply [21], certain hormonal imbalances such as Poly Cystic Ovary Syndrome may inhibit lactation [22]. This would mean that, despite lactation support, a mother may have a reduced supply.

## Method

A case study approach was adopted to consider the how and why questions, without manipulation of behaviour, and with consideration for the context in which it occurs [23]. There are many ways in which to research a case, and case study methodology is a creative way of binding a phenomenon through time, place, activity, definition, and context in an open and transparent way. Many theorists define and categorise types of case study in slightly different ways, and across all approaches the importance throughout is the consideration for the case, purpose, approach, and process of case design [24].

In this instance a key case was selected [23], characterised in this instance as a breastfeeding mother that is actively seeking donor breastmilk online. The process adopted in this research was a single diachronic study, or a type 1, holistic, single-case study [23], that utilises a descriptive and interpretative approach [25]. The descriptive elements of this approach concentrate on describing the different aspects of the phenomenon, whilst the interpretative approach explores how the phenomenon unfolds over time. The purpose of the study is one of exploration, like many exploratory studies, there is not sufficient knowledge on which to base any propositions, so the exploration is based on the purpose and aims of the study [26]. Given all experiences are assumed individual and subjective the exploratory design facilitates the understanding of the women’s story where outcomes are not predicted. The study choices are detailed in Table 1.

**Table 1:** Elements chosen within for the design of this case study [24]

Subject	Purpose	Approach	Process	Process detail
Outlier case	Intrinsic	Test theory	**Single**	Nested
**Key case**	Instrumental	Build theory	Multiple	Parallel
Local Case	Evaluative	Draw a picture		Sequential
	**Exploratory**	**Descriptive**		Retrospective
	Explanatory	**Interpretative**		Snapshot
				**Diachronic**

### Participant

Abbi was one of ten mothers who responded to an advertisement on the HM4HB Facebook group, in May 2019. All were eligible to participate in the study and presented a purposive single case worthy of exploration. All mothers were seeking donor breastmilk for their child, at the contemplation stage as a recipient, and had access to professional lactation support should they need it. Only those that had experience of the phenomena could take part. The exclusion criteria included any mother that did not satisfy the inclusion criteria, people seeking breastmilk for themselves, and those who were not seeking breastmilk for infant feeding purposes, and this study was not limited to first time mothers.

Abbi was selected as the chosen participant based on convenience, she was easily accessible, forth coming in making contact and asking questions, and eager and willing to share her story. Abbi was the first mother that was forthcoming, easily contactable, and willing to undertake this study over a period of months. Following an email from Abbi, a telephone call was undertaken to build a professional relationship and rapport [27], and to ensure that she understood that her story was the primary focus of the study, and research aims.

### Data collection

Abbi recorded her thoughts and feelings as she searched for donor milk over a two-month period (June and July 2019). This was received in the form of screen shots taken from her online chats with friends, and posts made to online social support groups. This aimed to capture the contemplative stage at that specific time, and to accurately describe a most comprehensive description of the case.

The main body of data was taken from a one-and-a-half-hour telephone interview (conducted in July, 2019), which was audio recorded and then transcribed by hand. The interview consisted of open questions that focused on the overarching research question [26]. Abbi was asked to firstly define and describe online milk sharing communities, discuss their use, and then to describe the meaning of the group providing examples, before reflecting on their use and what she would do moving forward.

Over a one-week period, at the time of the interview, all activity on the open Facebook group, Human Milk for Human Babies, was recorded. This is a publicly visible page and access is unrestricted. Page activity such as the number of posts made, content of the posts and subsequent comments were recorded to provide a context to the study and relate this to this mother’s journey. This data provides context to the case, sources of information independent to the case, and an opportunity to use many sources of evidence.

### Data analysis and write up

The analysis of the findings focuses on the mother’s experiences and does not aim to generalise any of the findings [28]. It focuses on the narrative description and pattern matching was adopted within the analysis to develop this case description [29]. In this instance data from the notes made by the mother prior to interview were matched to the narratives from the interviews to strengthen the case analysis.

A combination of linear-analytic and chronological structures was used to write up this study, characterised by the analysis presented in a linear, logical order and therefore complements the chronological approach to presenting data [23]. Hammersley’s quality criteria [29] for case studies was considered throughout the research process and a journal of thoughts was kept during the process and data collection also ensured that the case study was interpreted inductively [30].

Abbi was given the opportunity to read the transcribed interviews to ensure she was happy with the final account and to check that no mistakes had be made, through the process of member checking [23]. A second follow up call was arranged to discuss any discrepancies or alternatives required, providing an opportunity to ensure they had been accurately recorded and therefore credible.

### Ethics

Full ethical approval was granted through Canterbury Christ Church University Ethics Panel, and the research adopts a ‘thick disguise’ [31]. Informed consent was ensured, and the motives of the researcher are openly discussed along with the rights of the participant to withdrawn at any point up to publication [31].

## Results

Abbi is a 29-year-old mother of three sons; Logan aged nine, Harry aged six and Lucas aged four months. Abbi lives in the England with her husband and children and is of British nationality. She has a National Vocational Qualification in childcare and volunteers with the National Childbirth Trust providing mother support groups. In 2004, Abbi was diagnosed with polycystic ovary syndrome (PCOS), and then in 2009, after her first child she realised that her PCOS negatively affected her breastmilk supply. This case study however, shares Abbi’s attempts to breastfeed Lucas, and her efforts to obtain breastmilk through online milk sharing sites, to supplement her supply. In chronological order it explores the meaning behind seeking donor milk and using online milk sharing sites, whilst she reflects on the experiences with her first two children.

### The journey so far

At the start of the interview Abbi openly discusses her expectations around breastfeeding when becoming a mother for the first time:

*“For as long as I can remember I’ve wanted to breastfeed and it’s been the one thing I wanted to do as a mum was breastfeed, when I couldn’t do that, it I suppose it’s a very natural, it’s meant to be one of the most natural things in the world just to feed your baby that’s what breasts are for and when I can’t do that it felt like it was my fault like my body had failed him. Um I did find that quite hard to deal with … it was just something I wanted to do, I don’t know why I have no idea, it’s just something I wanted to do and expected to do … so I didn’t expect it to be as hard or even not produce milk to be able to feed my baby that was just a bit of a shock, that I didn’t have any milk. Yea I wasn’t expecting that”.*

Abbi breastfed her eldest for two days before switching to formula. She described a lack of milk and a lack of support around breastfeeding at that time. With her second child she again felt as though she had a limited milk supply but did feel more supported with her breastfeeding. Abbi openly discussed the impact that this had on her mental health and feels that it was a large contributing factor to the Post-Natal Depression (PND) she experienced with Logan and Harry. These were feelings that this time Abbi was keen to avoid and motivated her during pregnancy with Lucas to take a different approach.

### **A need for milk:***“It’s not just milk it’s mental health”.*

When pregnant with Lucas, Abbi felt determined to try to breastfeed, as she was not planning to have more children in the future, she felt as though this was her last attempt to fulfil her desire to breastfeed. This determination led to certain behaviours to make this into a reality. Even when pregnant she was looking for people, and surrounded herself with breastfeeding mothers who could help her visualise and achieve her goal to breastfeed Lucas:

*“So, I knew who to speak to when I needed help and I think that was a big help knowing where to go when I was struggling and needed support. I knew where to go for support”.*

Abbi also joined online breastfeeding support groups for support and information in the hope of breastfeeding and thus reducing the reoccurrence of PND this time around:

*“A big part of it [PND] was not being able to breastfeed because one of the things that I wanted to do was breastfeed and because I couldn’t I felt I failed them, so yea I was more determined his time as it was my last chance to breastfeed”.*

Since Lucas’ birth Abbi has been breastfeeding him, however this has been a turbulent time. The messages Abbi shared between her and a friend show just how difficult this time was:

*“Spent the day in hospital after Lucas lost weight again. He is down to 3320g. More than 11% loss. Got to keep feeding him every two hours, then express straight after then give him expressed milk … I’m finding it very hard to do more … I’m tired and emotional not sure if there is any point in going to bed tonight, will probably sleep on the sofa … by the time Lucas has fed, I’ve pumped, expressed milk and I’ve washed the pump up and sterilized it will be time to start feeding Lucas again. I really hope this works quickly as I can’t see how I can keep this up long term … I can’t physically pump anymore”.*

Abbi soon realised that her PCOS was affecting her milk supply. Her reaction was to seek both professional help, and support from the peers, to try and maximise her milk supply. In the interview Abbi said:

*“So, we have pumped, to start with I was pumping every two hours as well as feeding every two hours, um yea, and I have used fenugreek [A herb that has been seen to increase breastmilk production] and I’ve had medications [Prescribed medications for example Metformin or Domperidone that, as a side effect, have be seen to increase breastmilk production”.*

In an early message posted to an online support group, at the time when she began taking prescribed medication to increase her supply, Abbi felt a moment of joy when Lucas had gained weight, she wrote:

*“Basically, they like them to put on 35g per day [in weight], so 100g in 4 days. So, the fact he put on 155g in 4 days is epic. That was with 2-3 formula top ups of between 1-3 oz per day. Now going to max of two formula top ups per day and this is while he was feeding so managed to pump 60mls!!! Most yet! I also started taking Fenugreek and Domperidone yesterday so hoping that helps my supply”.*

Abbi describes how she pumped her milk regularly to increase supply, she took fenugreek and took a two-week course of Domperidone. This was prescribed off-licence by her primary care physician as a Galactagogue known to increase milk supply (Breastfeeding Network, 2014). Abbi worked with an International Board-Certified Lactation Consultant (IBCLC) and after a few weeks started on Metformin, which replaced the Domperidone prescription. Top ups of expressed milk were given to Lucas and a supplementary nursing system was also one of her final attempts in increasing her milk supply. In the post above, it is seen that Abbi used the online groups to share the joy of her successes within a community of breastfeeding mothers who would understand her efforts.

At around four weeks old, Lucas was not gaining weight. In messages to friends Abbi wrote:

*“Lucas has lost more weight. Paed [Paediatric] registrars want him re-weighing in 48 hours. Midwife has recommended that I express every other feed but top up if he is unsettled. Basically, it’s my low supply that’s causing the issues, so I’m feeling a little fragile/ guilty but won’t be giving up feeding”.*

Abbi’s sadness, yet determination, can be heard above This led Abbi to ask many groups for advice online, in the interview she said:

*“Yea it’s a big decision [seeking donor milk], he was low weight gain because of my low supply, and we were having Midwife and Health Visitor visiting every two days and we were having admissions because of low weight gain. They were trying to convince us that formula was the way forward and although he went on formula for about a week, he still didn’t put any weight on, he just became constipated from it, and it was almost out of desperation that I needed to do something, just to help. It was almost out of desperation that I put that post up thinking no one going to help us, but I have to try something this is not working for us and I need to do something, so I did”.*

The desperation that Abbi talks about in her interview was seen in messages that she had posted online: *“What can I do to increase my low supply? Lucas has lost more weight”.* This was a difficult time for Abbi: *“um I started to worry that I wasn’t good enough and I could feel them doubts and failure creeping back and I thought I don’t want this, and I decided to do something”.*

At 4 weeks old Lucas was supplemented with infant formula. Abbi believes that as a direct consequence of this Lucas became constipated and unsettled and Abbi describes feeling desperate as she began viewing the infant formula negatively. Abbi talks of how she did not like the effects of infant formula on her baby, or the impact of not feeding on her mental health.

It was at this time that a close friend offered her some of her own frozen expressed breastmilk and she accepted. She began looking more into donor breastmilk as an option for Lucas, something she had not considered previously. This avenue of support Abbi feels was due to her own internal issues as opposed to pressure from professionals or others to breastfeed stating *“it’s not just milk, it’s mental health”.* Abbi stated that breastfeeding could be promoted more and talked of her own internal thoughts that caused her to search for donor milk online.

Following a need for support, Abbi went online. She talks of the benefits of online support groups in general, in providing options and ideas from others, support not available in offline communities. The value of the online groups in providing access at all times of the day, to encouraging words and information was noted. There was a felt need to find milk, and a real tangible need.

*“Um I’ve posted in the middle of the night asking something really silly, but checking like am I doing this right, should I keep going, should I stop, is he getting enough milk um unfortunately in this case I wasn’t, but I was signposted to places that helped, and it showed me how it helped me see that he’s getting enough milk … and I posted on the group about milk donation … just loads of different things … I don’t feel like there is pressure to breastfeed … it was my own guilt and my own feelings it wasn’t because of what somebody else had said. It was my own issues that made me feel guilty”.*

From previous children Abbi already had an online group of friends. They had originally met in a parenting group; however, the group implemented an increasing amount of group rules, so a select number of mothers started their own group, a group of likeminded people:

*“Most of the group breastfed to term [Breastfeeding until the child no longer asks for breastmilk], cloth nappy and use baby carriers and are known as alternative/ gentle parents. I love the group for its honesty and help. They all tell me straight if I need to get a grip or if I should be doing something differently but are also the most supportive strangers I have ever met”.*

It was interesting to hear how easily these online groups form, and continue over several years, yet Abbi still referred to them as strangers because they had never met in person. She had strong ties to this group of mothers and showed a sense of agency in her online support behaviours. She knew where to go and where not to go for support around donor milk. Abbi shared some messages between her close friends who were long time breast feeders stating, *“I messaged them as I knew they would be supportive and caring”*. Abbi liked to share her successes with her online groups and received many words of encouragement in reply. Typically, the small weight gains that Lucas achieved were points of celebration for Abbi and she shared these with the group *“60g weight gain!!!, 155g weight gain in 4 days!!!!, I am loving our breastfeeding so want to continue, I am so proud of this one whole week!!!”.*

### Finding a donor

When Lucas was around four weeks old Abbi discussed milk donation within an online breastfeeding support group. She discussed how some sites were not supportive of these discussions and had a blanket ban about talking about milk donation, leaving her feeling judged:

*“Some of the other groups that I am on have a blanket ban on it not being talked about at all. I was told because it was unregulated, even when I said about it from the milk banks, they had a blanket ban and say no we don’t talk about, we don’t have milk donated in our group. I left that group quite quickly because no I didn’t understand that at all … I found that very judgemental and I did leave. If that has been the only group that I was on I would have found that very difficult to carry on breastfeeding because I didn’t have the support there … it’s a shame that they took that view. I could stay in the group I just couldn’t talk about; I wasn’t allowed to talk about donor milk on there. It made me feel judged and it did make me feel like a failure that I was having to use donor milk to help my baby... um it’s still breastmilk and yea I did I took deep breaths and thought right well that’s how they feel get rid of the group and I don’t need them sort of people in my life I can go to my other group and say help me and that’s what they did, they then did help”.*

Abbi showed her sense of control over her online behaviours. She demonstrated her ability to know how, where and where not to seek support, *“so I looked into it and researched it”*, using the information from peers and from professionals to meet both her own and her baby’s needs. Abbi discussed how she valued the science provided by professionals but values the experiences of others when the science did not work for her. Abbi discussed making an informed choice and her thought processes before using donor milk facilitated by an online milk sharing site. She weighed up the risks, was aware of the risks of peer shared milk, and developed her own internal criteria before using peer led donor breastmilk.

After searching Abbi found the HM4HB page, a peer-to-peer human breastmilk sharing site stating, *“Once I’d found the right channels it was easy to access”.* This social media group linked Abbi to similar minded people that she had not met before. Via the site, and when Lucas was 5 weeks old, she successfully found a breastmilk donor.

This tangible online support elicited many feelings for Abbi. On one hand Abbi described feeling joy:

*“Just amazed, just... I was amazed that people do that for other people, amazed about it but also gutted that I didn’t know about it for my other children because it would have made such a difference to how I felt after. Because I was diagnosed with post-natal depression, I think it would have really helped me feel not as low as I did in those times, because I would have still given them breastmilk which to me is preference over formula”.*

Abbi also experienced feeling *“saved”:*

*“Oh goodness someone has helped us, we haven’t got to use that formula, he won’t get belly ache any longer because he was really struggling with constipation and I was like this formula isn’t working and he can have breastmilk, I can keep breastfeeding him. Um, it saved my sanity and my mental health, yea I was overjoyed completely overjoyed, overwhelmed, overwhelmed by somebody else’s kindness, and it means my baby is healthy and thriving and happy”.*

Such positive feelings also brought with them some apprehensive, as she was nervous about taking milk from a stranger:

*“I was very unsure, not unsure about it, just really, I don’t know apprehensive about it I suppose about somebody else’s breastmilk, but I thought it was better than what we were going through now … but at the time I was a bit apprehensive about it to start with, quite nervous about accepting somebody else’s breastmilk I suppose I worried that I didn’t really know, wasn’t sure if I really knew the person. Um, but I made sure I looked into who was donating and actually met them before accepting the milk. When I started thinking about it, it was more of ugh I was apprehensive but also very much a last resort, but now I’m very positive and I’ve told people about it and promote it when I can”.*

Here we can see a change in Abbi’s thinking, from once nervous emotions to a confidence in what she is doing.

### Group activity

At the time of interview, the human milk for human babies’ site had 22,281 followers on their UK page. To provide context the group was created on Facebook on 28.2.11 and year on year the group attracts around 2000 new followers to its pages. On 20.12.17 the page had 18,000 members and then by the 7.9.18 had 20,000, hitting 21,000 members on 21.1.19 and then 22,000 by March.

During the week of the interview (July 2019) there were 55 posts to the page. Just twelve of the post were from donee’s, like Abbi, requesting milk, which were then reposted by administrators 11 additional times. The page also had 16 mothers come forward offering donor milk, which administrators shared 16 additional times to increase visibility of the posts. #milk to share, #freezerstash, #milktoshare, #donormilk were all common social media tags added to increase visibility to these posts.

On the site, the posts from donors were typically short detailing amounts and location of donor milk, for example *“I have 40oz of dairy free milk pumped on Friday in …*” *,* or *“70oz to donate in [area name]. Pumped from December up to March. Looking for a happy home”.* Most posts also contained a picture. These posts are easy to read, and it is easy to identify which would be appropriate in terms of location.

### **Meeting the donor:***“Trust not regulation”*

When Lucas was about three months old Abbi met Lauren, a milk donor through the HM4HB site:

*“She posted on a local group um saying she had some and I was tagged in the post by several different people, and I messaged her we messaged each other for a little while as we talked”.*

Abbi describes talking with Lauren, asking open questions, and building a relationship with her. She discussed lifestyle habits such as alcohol use, smoking, diet, and medications until she felt comforted and reassured:

*“We talked before and she was very open about everything that she had been through um how many children she had and the whole background which made me feel happier about doing, about accepting the milk. I suppose if somebody wasn’t very open or preferred not to discuss certain areas that I did ask about, like smoking and drinking. Because those things would put me off because I don’t do those things, or if they were on lots of medications, I think at the start that would have put me off”.*

Abbi discussed her reasoning:

*[The decision to use donor milk] “It was based on they wouldn’t feed their own baby with that milk if it could harm them, so why would they give it to my baby … I suppose it helps my trust that other people know her, um that she’s local because Lucas doesn’t like being in the car. I prefer someone who’s friendly, um and I can speak to them freely and they can speak to me freely. I think if someone was abrupt or not very open or I don’t feel that I wouldn’t then trust them if that makes sense … it a relationship definitely, that mother is feeding my baby basically and I feel that relationship, and friendly relationship yes”.*

Abbi did obtain donor breastmilk from a lady that she did not feel she made a personal connection with. Despite collecting the breastmilk, she did not feel that she wanted to use it. Abbi described how she and Lauren had friends in common and frequented the same group which made her feel at ease about this donation.

Abbi also sought support from a milk bank. In the early days Abbi was unaware that she could get milk from the milk banks, writing to a friend:

*“I’m using donor milk from a local friend but running low so will probably need to use formula again soon bit it made him horribly constipated before. We have started the process of applying to the milk bank, but I doubt I will get funding or accepted as we are low on the order of need”.*

Abbi received two batches of milk via the milk bank which was delivered straight to her house. She believes that *“breastmilk is precious and shouldn’t be wasted”* and because the milk bank donates to the hospitals, she was happy to also receive milk from peers because it would mean there was plenty of milk to go around:

*“I definitely feel that even when I had Lucas and even when I had Harry there was only mention of NICU [Neonatal Intensive Care Unit] having to have the milk, and it wasn’t available anywhere else and even when I had Lucas this time, there was still a thought that it was only really available to NICU’s and poorly babies, um and it was hard to access which although I had to find the right channels, once I had found those channels it was easy to access. Once you’re in a breastfeeding group it is very easy to find out about it”.*

Through the online support groups Abbi has been able to find a regular donor Now that she has a regular donor, she feels she doesn’t have to use this form of tangible support at the moment.

*“I use the local online group the most, and the donor milk one I suppose it’s the HM4HB group I was using a lot, but I’ve got a regular donor now so I’m not having to use it. Well one lady she had four moths supply in her freezer and what she can pump in a month is just under what Lucas needs in a month, so I’m not having to use it”.*

### **Impact of the online milk sharing group on breastfeeding journey:***“it’s not just babies’ wellbeing it’s mums’ wellbeing”.*

Abbi used the milk sharing site and an online breastfeeding support group to support her journey. Both contributed differently to her journey and this case provided valuable insight into how different groups can support in different ways, and the diversity in groups. Overall, Abbi feels social media has made breastfeeding support and particularly donor milk more accessible:

*“I suppose it’s, it is social media that has made it more, more like, accessible to me, I thought in our area it was just for NICU and new babies … because it’s there all the time, someone to encourage to say keep going you’re doing well, which in the middle of the night is vital”.*

She found vital support for her breastfeeding journey through the online support groups:

*“The online groups have been a life saver in the middle of the night, yea it is, it really is mental health, and it is just a lifesaving support group. It probably doesn’t sound like it, but it really does help with the worry and concerns and just the loneliness in the middle of the night when you are struggling. It’s there. You don’t have to post something you just have to read through what other people have written and make sure like you’re not alone you’re fine carry on going, un, yea it has the breastfeeding group has made a big difference and impact in my life to keep going to where we are now and hopefully keep as long as we can”.*

The main reason given was the access to esteem support and words of encouragement throughout the day and the night that have kept Abbi’s motivation high and supported the longevity of feeding. Due to Abbi’s previous history with unsuccessful breastfeeding the group has been vital to her:

*“The online group is there all the time. There is always someone there to answer your question, or if you’re having a rough night there’s always someone there to go keep going, don’t stop just make sure you’re happy and baby’s happy … in the middle of the night it’s vital”.*

The informational support and access to both professionals and peers was also indispensable to Abbi:

*“I looked into it and researched it and I asked several groups about it and um and yea there were lots of people that were willing to help and then I was signposted to other people on other groups and organisations that so it as professional channels as opposed to my friends … another benefit of the online group is loads of people have loads of different tips and they can tell you about donor milks and what can happen”.*

Abbi described how the HM4HB site links her with donors *that “have had bits and bobs of what people can donate”*. Due to Abbi’s own fluctuating supply, she has relied on these bits and bobs from peers and the milk bank, using formula as a last resort. She talks of fluctuations and growth spurts and the uncertainty of this process. One message in the early days, to her online group, read *“My supply seems to have dipped again so I am waiting to hear from the milk bank about the next delivery as none left from them”.* The impact of this for Abbi meant that she set short-term and long-term breastfeeding goals. Abbi’s long-term goal is for Lucas to self-wean from the breast, but she talks of having enough human milk to support Lucas until he is eight months old. Abbi adds that if something were to happen now, she feels she would know that she has done her best and would not have the troubles with depression that she has previous had because of this:

*“It means the absolute world to me and saved my sanity and my mental health … I’m now feeling like if something happened this week and we had to stop I feel like I have given it my best shot and it wouldn’t affect me as much as not feeding. I don’t feel like a failure … The online groups have provided professional and peer support and feels as though all the groups are about being there, experience, knowing how it affects you and being there”.*

Abbi believes that both groups could certainly help other mothers going through the same thing as her:

*“Now I’m very positive and I’ve told other people about it and I’ve told them to look into it and I promote it as and when I can it’s the most amazing things to do and it helps so much. It’s not just baby’s wellbeing it’s for the mum’s wellbeing., my mental health has been so much better this time”.*

### Future wishes

Abbi wanted to share her story to encourage others to seek support online to meet their breastfeeding goals. She wanted to share the difference that she feels it has made to her and for her son Lucas. She feels that donor milk needs promoting, and an awareness needs to be raised about this so that other mothers can make informed choices:

*“I didn’t realise there were people who were willing to do it and I could access it through a milk bank … professionals need to talk about it and provide leaflets and information … I think it’s such a small area of Facebook and social media that know about it and only a small amount of breastfeeding mums that know about it as well, but it seems to be getting more popular as its being promoted and made awareness of, but I think it depends on, it’s sort of quite blurry because not all women can feed, can pump and express their milk to do it. It is only what is available. Um so it is there but a very small select people can donate their milk”.*

Here Abbi shows the power of social media in linking this small group of women with other donors and donee’s.

Abbi also wanted to share her story to encourage professionals to talk to mothers about all available options and to be aware of the milk sharing sites saying, *“I do think more professionals should be aware of milk banks and they should promote them, or they should have information to hand just like they would have information to handout about another other medical, because it is a medical need as much as, my baby needs feeding”.* Abbi felt as though her health visitor didn’t want to talk about milk sharing or the use of milk sharing sites stating, *“she was definitely scared as a professional to talk about it, it felt she would have preferred not to talk about it at all”.* This has implications for future practice as she felt she would have benefited from talking about this with a professional, or even an awareness week, *“it needs promoting we need an awareness week. We need to get this subject out there and who other people that its possible and it does help and it’s more than just food … we need social media for things like this”.*

## Discussion

This is amongst the first studies to provide an in-depth exploration of a mother seeking donor breastmilk online. Abbi’s case goes part way in exploring how and why the phenomenon of tangible online social support groups could support the breastfeeding mother. The HM4HB site, during the week of the interview, saw twelve mothers, like Abbi, searching for donor milk. The group provides clear posts from people wanting to receive donor breastmilk and people wanting to donate, in the hope that the two will link up and exchange can occur. Although tangible support is evident through how the group works, it was the deeper cognitive processes, highlighted within this study, that were enlightening.

Abbi described how Polycystic Ovary Syndrome reduced her milk supply, something noted in the literature [22, 32], and how not breastfeeding significantly contributed to her depression. Research has shown that experiences of lactation insufficiency can be extremely difficult, isolating and cause a ‘breastfeeding grief’ that often goes unnoticed [10, 33]. This is a time when mothers need all types of support [34]. The research also offers a discussion around how the group provided hope, along with options and ideas. The ability of social support to support a person’s mental health, reduce stress and increase a person’s resilience is well noted [35–38], and unique to this study is the contribution of tangible online milk sharing social support groups in supporting breastfeeding mothers.

There were several points worthy of discussion, and one area that echoed through the research was the stigma around milk sharing. Despite being a historical practice, still widely practiced today in developing countries, the stigma around milk sharing in the UK is noted in this study through the lack of discussion offered to Abbi around the use of donor milk by the professionals involved in her care, the fact the online breastfeeding groups that support breastfeeding banned such conversations, and from Abbi herself who chose who not to disclose her milk sharing with, through fear of being judged.

Stigma around milk sharing amongst health care professionals leads many women to do so in secret. Professional’s fear saying the wrong thing, losing their jobs and loosing face in communities are some reasons why such stigma exists [10]. This research notes Abbi’s impression that the professional did not want to talk to her about this topic. Health professional who actively involve themselves in an informal exchange of milk can raise both ethical and liability questions [21], which has implications for future practice.

This research has highlighted a source of information online that mothers could be signposted too, however open, and honest discussions should be had between the professional and mothers. Abbi felt that if she had had conversations with professionals then it may have made a real difference to her breastfeeding journey, and felt social media was a platform to raise awareness. Similarly, women feared talking to professionals through fear of stigma or even being refereed to child protective services [10].

The professional’s advice given to Abbi is also worthy of discussion. Poor supply, and Lucas’ subsequent slow weight gain, meant that professionals advised Abbi to introduce some infant formula milk after feeds, which she did. Formula milks are often used, unquestioned, expected within a predominantly bottle-feeding culture, and a convenient alternative to a mother’s own milk [10]. Professionals in the UK follow the National Institute of Clinical Excellence (NICE) guidance on the recognition and management of faltering growth in children [39], and the NICE guidance warns that whilst supplementary feeding with formula may increase weight gain in the breastfed baby with faltering growth, it often results in cessation of breastfeeding. As Abbi experienced, no donor milk was offered in or outside of the hospital setting, despite it being available through regulated milk banks.

Abbi demonstrated a sense of agency navigating through different types of online support groups, engaging with those where she felt supported to meet her gaols. Although HM4HB simply links donors with donee’s, psychologically it provided hope, and allowed Abbi to feel as though she was doing her best. This was coupled with the reassurance and esteem support from other online groups which bolstered and protected her mental wellbeing. Abbi stated her PND would have been worse if it was not for the group, through the many options that the group provides.

Overall, on one side, breastmilk is idealised, however on the other, milk sharing can provoke stigma and thought to be dangerous. Health professionals may avoid conversations that conflict with guidelines; however, this causes people to search online and hide their practices, preventing opportunities for discussion and honest conversation to ameliorate risk. Health professionals must help explore options, making sure they have the information and advice that is evidence based.

### Study limitations

Case study approach has often been criticised for lacking scientific rigour and providing little basis for generalisation [23]. Case study is also dependant on the wider political and social environment. These results present one case, one example of tangible online breastfeeding support offered through the milk sharing site, and these results have not been generalised. Alternative explanations around the use of these tangible online support groups are possible.

## Conclusion

This is a case of one mother’s journey to provide breastmilk for her child, facilitated through online peer to peer milk sharing sites. Recognising negative feelings and to prevent occurrence of mental ill health, Abbi sought online support and came across donor milk sharing online. The sites, although when found were easy to use, and offered tangible support, there was much deeper psychological meanings and realities expressed including guilt, mental ill health, and necessity. This was not simply tangible support, and Abbi dealt with the decision making around donor milk on her own. Labelling the group as tangible support ignores the complexities around milk sharing.

For Abbi she felt she needed to build a trusting relationship with the donor first. At the beginning, the feeling of joy and feeling *“saved”* were also coupled with anxiety around the potential risks that she was fully aware of. When these relationships with the donors were forged Abbi found the process to *“mean the absolute world”* to her and she wanted to share her story with others to promote online social support for breastfeeding women. Abbi talks positively of the HM4HB site and the positive impact that this tangible support had on her breastfeeding journey and her mental health.

Abbi’s journey is a good example of how online social support can support an individual’s breastfeeding journey. The HM4HB site, although tangible in nature, links donors with donee’s and fulfils a much greater need than simply providing breastmilk. Abbi states *“it’s more than just milk it’s mental health”.*

More awareness of the availability of donor milk and peer to peer sharing online is required. Looking at the online environment there are thousands of mothers who are doing the same, having to navigate the online environments with varying levels of professional support to aid their decision making. Professionals working with new mothers and babies must ensure they have the evidence and skills to have these conversations so that they can best support the mothers and infants that they work with. All professionals with a role in breastfeeding support should have the courage to engage in difficult conversations that reduce any perceived stigma, at the same time as providing evidence based and reliable information so that mothers can make informed choices, maintain their own autonomy and make their own decisions.

## Data Availability

The datasets used and/or analysed during the current study are available from the corresponding author on reasonable request.

## Abbreviations

HM4HB: Human Milk for Human babies
IBCLC: International Board Certified Lactation Consultant
NICE: National Institute for Health and Care Excellence
NICU: Neonatal Intensive care unit
PCOS: Poly Cystic Ovary Syndrome
PND: Post Natal Depression
UK: United Kingdom
